# Effects of embedded acceptance and commitment training in veterinary medical and technology programs as students transition into the workforce: A study protocol

**DOI:** 10.1371/journal.pone.0334898

**Published:** 2025-11-11

**Authors:** Mary Beth Spitznagel, John Martin, Nikki E. Bennett, Christopher Was, Christopher M. Fulkerson, Jim Weisman, Luca Giori, Erin Kelly-Snyder, Alanna S. G. Updegraff, Michael P. Twohig, Michael E. Levin

**Affiliations:** 1 Department of Psychological Sciences, Kent State University, Kent, Ohio, United States of America; 2 College of Veterinary Medicine, Purdue University, West LaFayette, Indianna, United States of America; 3 College of Veterinary Medicine, University of Tennessee - Knoxville, Knoxville, Tennessee, United States of America; 4 Veterinary Technology Program, Columbus State Community College, Columbus, Ohio, United States of America; 5 Department of Psychology, Utah State University, Logan, Utah, United States of America; PLOS: Public Library of Science, UNITED KINGDOM OF GREAT BRITAIN AND NORTHERN IRELAND

## Abstract

Psychological distress is elevated in the field of veterinary medicine. Recent evidence demonstrates that stress and burnout in veterinary workers can be reduced through an Acceptance and Commitment Training intervention targeting “burden transfer,” or reactivity to challenging interactions with veterinary clients (“Unburdened”). Exposing students to Unburdened could optimize mental health outcomes as they transition into the veterinary workforce. Unburdened will be adapted for student use in a self-guided digital format and embedded into 5 veterinary medical or technology programs. Participants will be 200 advanced students recruited into a parallel-arms design: assessment-only Control versus Intervention (<2 hour Unburdened intervention embedded into final semester requirements) conditions. Online assessments at baseline, program completion (1 month), and follow-up (3, 6, 9, 12 months) will measure Kirkpatrick outcomes: Reaction (engagement, perception of the program), Learning (knowledge test performance), Behavior (skill use frequency), and Results (burden transfer, stress, burnout, anxiety, and depression). Latent growth curve modeling will compare conditions throughout the year following graduation. Institutional Review Board approval has been obtained. Informed consent is electronically granted by students electing to participate. A safety officer unconnected to the study will monitor study progress and safety. Pending positive outcomes, this ready-to-share program will be made available to training programs. If successful, its routine adoption into training curricula could decrease burden transfer, stress, burnout, anxiety, and depression in the field. Ultimately, the proposed work could provide a scalable and sustainable strategy to enhance mental health and well-being across the veterinary workforce.

**Study registration:** The trial to evaluate Unburdened’s potential impact on mental health is considered observational, as the involved educational institutions chose to implement the program for its educational value. Details can be found in NIH RePORTER: https://reporter.nih.gov/search/FhAMx6slrEautC8YKesk0Q/project-details/10980628. This study is registered with the Open Science Framework, where data will ultimately be made available: DOI 10.17605/OSF.IO/QEAKM.

## Introduction

While highly rewarding in many ways, occupational hazards of veterinary medicine can make it an emotionally challenging work setting [1–5]. Despite the urgent need to ensure wellbeing in this field, reviews of wellness interventions in veterinary healthcare providers note a lack of rigorous research [6] and call for evidence-based approaches [7]. Focusing on mechanisms of distress associated with specific and systemic problems in the field may be valuable.

One driver of distress in the field is a high frequency of difficult client interactions [8,9]. “Burden transfer” occurs when the distress of veterinary clients about their companion animal leads to challenging interactions with veterinary providers, shifting distress to the healthcare team [9]. This transfer of burden has been recently identified as a potential mechanism of occupational distress in the field of veterinary medicine [9,10]. Notably, evidence suggests that while frequency of difficult client interactions is related to stress and burnout, reaction to the situation is a far stronger predictor of these negative outcomes [9]. Therein lies a mechanism for change, and strong potential for improvement.

Recent research demonstrates the usefulness of “Unburdened,” an Acceptance and Commitment Training (ACT)-based intervention, in helping veterinary healthcare teams alter their response to challenging client interactions [10–12]. ACT teaches self-reflection through mindfulness and acceptance interventions via experiential exercises, while providing techniques for behavior change [13]. Meta-analysis of ACT in the workplace shows medium effect size reductions in stress [14], with recent work demonstrating significant reductions of distress and burnout in healthcare staff [15]. Moreover, this modality is effective even when delivered in a digital self-guided format [16], which provides a highly scalable and accessible format for disseminating ACT. Unburdened, with its focus on difficult client interactions in the veterinary healthcare setting (see Methods for greater detail), has exhibited effectiveness across various delivery methods (e.g., small group, telehealth, remote asynchronous) in enhancing mental health outcomes for persons currently working in this field [10–12].

Used in a training setting, Unburdened is likely to facilitate important competencies identified in the veterinary educational frameworks [17,18], such as skills for reflecting on personal actions, promoting attention to sources of workplace stress, and taking action to remedy adverse situations. Additionally, students preparing for a career in veterinary medicine are already likely to be experiencing emotional distress: 49% report at least moderate stress, 66% report mild to moderate depressive symptoms, and 30% are at high risk for burnout [19–21]. A tailored-for-students version of Unburdened, developed to be broadly adopted into curricula of veterinary doctorate (DVM) and veterinary technology (RVT) training programs, has the potential to enhance well-being and reduce the likelihood of future stress and burnout for future generations of providers.

The overall aim of this project is to study whether Unburdened training, adapted for student use (“Unburdened – Student Edition”) in a digital self-guided format and embedded into DVM and RVT education, can reduce stress, burnout, and symptoms of depression and anxiety as trainees transition into the workforce. Pending successful outcomes, the easily shareable program will be made broadly available for DVM and RVT programs.

## Methods

### Study design

This project is a parallel arms study recruiting participants from 5 training programs agreeing to embed the intervention into final semester requirements on an alternating basis. Site alternation was chosen over individual randomization to prevent risk of students within tight-knit cohorts sharing Unburdened – Student Edition techniques with Control students. Unburdened – Student Edition will be completed at a pace of 1 session per week (approximately 30 minutes per session) over the course of 3 weeks. During years in which the program is embedded at an institution (Intervention), online research assessments will be conducted at baseline, completion (1 month), and follow-up (3, 6, 9, 12 months). When the intervention is not embedded at an institution, participants will complete measures at the same intervals (i.e., “Training as Usual” Control). The intervention will be made freely available to controls after their participation in the study is completed. See Table 1. Enrollment began March 17, 2025. Assuming the study enrolls as expected, the anticipated end of recruitment will be May of 2027, with an anticipated end of data collection in May of 2028. Peer review of the protocol was conducted as part of the federal review process of the National Institute of Safety and Occupational Health (Safety and Occupational Health study section), as well as by an independent safety officer consulting for the study. The primary institutional review board reviewed and approved the protocol; participating institutions reviewed the protocol and deemed reliance upon the primary IRB appropriate.

**Table 1:** Schedule of enrollment, intervention/control, and assessments for each cohort.

	Enrollment	Allocation	Post-allocation							Close-out
TIMEPOINT	*-t*_*1*_	0	*Week 1*	*Week 2*	*Week 3*	*Week 4*	*Week 13*	*Week 26*	*Week 39*	*Week52*
**ENROLLMENT:**										
**Eligibility screen**	X									
**Informed consent**	X									
**Allocation**		X								
**INTERVENTIONS:**										
**Control**										
**Intervention**			X	X	X					
**ASSESSMENTS:**										
**Demographic/ Background Information**	X									
**Hours Current Clinical Work**	X					X	X	X	X	X
**Reaction**	X		X	X	X					
**Learning**	X					X	X	X	X	X
**Behavior**	X					X	X	X	X	X
**Results**	X					X	X	X	X	X

The above timeline is repeated for each graduating cohort recruited, with 3 cohorts planned.

### Study setting

Study procedures all take place through the Kent State University’s Department of Psychological Sciences. Programs utilizing the Unburdened – Student Edition program and allowing student recruitment for research participation include Purdue College of Veterinary Medicine DVM and RVT programs, University of Tennessee – Knoxville DVM program, Columbus State Community College RVT program, and Kent State University RVT program.

### Ethical considerations

The protocol described here was approved by the Kent State University Institutional Review Board #1530. The project will be completed in accordance with the Declaration of Helsinki. The informed consent document is presented online prior to the online research survey opening; consent is documented through the participant clicking to agree to study procedures. Students are explicitly informed that participation in research is voluntary, can be discontinued at any time, will be kept confidential from their own educational institution, and that their decision to participate (or not) will not affect their grades or status in their program.

### Recruitment

The above institutions will embed Unburdened – Student Edition into final semester requirements as applicable (e.g., coursework, competency checklist) on an alternating basis, allowing for collection of both Intervention and Control data from all sites. Because the program is embedded into training, participation in research involves only the commitment to complete brief online questionnaires at 6 time points and allowing researchers to examine program engagement. Control participants will be recruited using pre-approved language sent by an instructor at their institution as a mass email containing the study link and informing students of the opportunity to participate in optional online survey-based research examining the “wellbeing of students as they transition into the workforce after graduation.” Interested students clicking the link will be directed to the Informed Consent, where they may opt in or out of research. Intervention sites will similarly inform students of an upcoming research opportunity via mass email; however, Intervention participants will be passively recruited through Unburdened – Student Edition. Upon enrollment, they will be informed that there is an optional research opportunity related to their use of the program. If the student clicks to “learn more,” they are directed to the Informed Consent, where they may opt in or out of research. Additionally, separate Control and Intervention site flyers have been created utilizing similar verbiage to the respective mass email messages; these will be placed in student mailboxes or distributed in class as applicable. Recruitment is expected to continue through April of 2027.

### Participants

Inclusion and exclusion criteria were chosen to maximize generalizability to student makeup of DVM and RVT programs, and ultimately to the workforce in veterinary medicine. Participants will be neither screened for nor excluded on the basis of common psychological disorders including depression and anxiety [22], as their exclusion could limit generalizability of findings; however, data collection and analyses will examine presence of common psychological symptoms including depression and anxiety. Inclusion criteria will include DVM and RVT students age 18 or older who are proficient in English and are planning to be in the veterinary workforce within 1 month of graduation. Exclusion criteria will include having less than 10 hours per week of direct interaction with veterinary clients.

### Intervention

The ACT framework is a well-established modality that relies on six interrelated components [13,23–26]. 1) “Being Present” involves consciously experiencing internal and external events in the moment. 2) “Acceptance” is the active embracing of inner struggles or difficult processes (e.g., thoughts, feelings) while they occur. 3) “Defusion” involves reducing excessive entanglement in thoughts by responding to thoughts as just thoughts. 4) Understanding of “Self-as Context” involves observation of inner experiences from an outside perspective. 5) “Values” are areas of importance to an individual that can be identified and embraced to guide action. Finally, taking 6) “Committed Action” means behaving with intention, in a manner that serves a chosen value.

The Unburdened program is based on these six processes, tailoring education and use of techniques to common challenging client interactions in the veterinary healthcare setting. Participants of the Unburdened program individually identify areas of burden transfer with which they struggle and learn these skills within a personalized framework. For example, a participant who experiences frustration associated with clients who repeatedly call for reassurance could learn to openly disentangle and observe their stress, connect with personal values of helpfulness and self-care, and commit to working with this client in a functional way. The protocol for Unburdened has been developed through a series of feasibility studies, randomized pilot trials, and randomized controlled trials examining it in various delivery formats (e.g., in-person group format, teleconferencing delivery, and asynchronous delivery). Through this iterative approach, the program has been refined into a digital self-guided format; this format has been adapted for student use (“Unburdened – Student Edition;” see **Table 2**).

**Table 2:** “Unburdened–Student edition” content.

*Week 1*
Introduction to client burden and burden transfer DANCE Identification of how points of burden transfer relate to difficult thoughts and feelings during DANCE interactions Introduction to being present, grounding and anchoring exercises, techniques for noticing the “here and now” Commitment to daily practice
*Homework: Daily practice of grounding/anchoring, noticing DANCE interactions, points of burden transfer, and challenging T/F
*Week 2*
Introduction to acceptance Tools to acknowledge thoughts and feelings in DANCE interactions without trying to control Introduction to defusion; imagery tools to allow thoughts and feelings to come and go Introduction to self-as-context; techniques for stepping back and observing thoughts and feelings Introduction to values; identification of personal values related to veterinary medicine
*Homework: Grounding during DANCE interactions, utilizing acceptance/defusion skills, noticing own values in clinical work and DANCE interactions
*Week 3*
Identification of individual derailed values in DANCE interactions Identification of individual ways to focus on alternate values and opportunities to bring derailed values back into life Introduction to committed action and individual ways to take values-based action Choosing a value to focus on in a specific DANCE interaction, identifying specific goals serving the value, and concrete actions to align
*Homework: Grounding, utilizing acceptance/defusion skills, noticing value derailment in DANCE interactions, acting in accordance with values

All components of this program are integrated into a single, Qualtrics-housed program that is easily shared and embedded into a learning system via a single link. Unburdened – Student Edition includes 3 main modules lasting approximately 30–45 minutes, each demarcated with a “halfway point” to maximize use in the classroom (i.e., program could ultimately be used during 3 classes at 1 session per class, or over 6 classes at ½ session per day to accommodate group discussion). Unburdened – Student Edition is a highly interactive tutorial involving brief passages presenting new material, student thought/responses, and experiential exercises, ultimately teaching ACT skills through constant input that integrates the individual’s personal experience. Accompanying the tutorial are frequent videos of recent DVM and RVT graduates sharing real-world workplace experiences involving burden transfer to help students, who may be less familiar with how client interactions impact the veterinary setting, authentically understand these dynamics.

### Assessments

Outcomes will be examined through objective and subjective measures utilizing the Kirkpatrick framework [27] to assess the degree to which participants respond favorably to the intervention (“Reaction”), knowledge demonstrated from it (“Learning”), extent to which their behavior reflects application of the new knowledge (“Behavior”), and degree to which the program results in targeted outcomes (“Results”). Measures will be assessed at each study timepoint (excepting basic demographic information which will only be collected at baseline, and Reaction variables, which can only be assessed following program use). Responses will be collected via Qualtrics and automatically scored to reduce potential error or bias in scoring.

#### ‘Reaction’.

Objective tracking of program use in Qualtrics will be conducted by examining percent of each Module that participants complete. Total percent will be examined to assess engagement. Subjective assessment of usefulness and relevance will be assessed via items presented on a Likert-type scale as in prior work [11,12], with assessment of internal consistency prior to analysis.

#### ‘Learning’.

Objective assessment of information learned in the intervention. Prior work [12] shows good internal consistency (α = 0.73) of this performance-based measure.

#### ‘Behavior’.

A frequency count will be taken of intervention skills used by participants estimating the frequency of use of learned techniques over the 2 weeks prior to assessment. Number of hours worked will be accounted for to produce average workday skill use. Past work [11,12] using this rating scheme shows excellent internal consistency (α = 0.92).

#### ‘Results’.

Previously validated self-report instruments will be used to examine outcomes, including the Burden Transfer Inventory (BTI) [9], Perceived Stress Scale (PSS) [28], Copenhagen Burnout Inventory (CBI) [29], Center for Epidemiology Studies Depression scale (CES-D) [30], and Generalized Anxiety Disorder 7-Item Scale (GAD-7) [31].

On the BTI, participants rate Frequency of and Reaction to 5 domains of DANCE interactions: Daily Hassles, Affect, Non-Adherent/ Inconsiderate, Confrontation, and Excess Communication. This measure demonstrates excellent internal consistency (α = 0.92-.94), respectively. The Reaction subscale will be the primary scale of interest, as it correlates strongly with stress and burnout (r = 0.39–0.70) [9]. Because Frequency may change in the transition from training to the workforce, this subscale will be used as a control variable in analyses if needed. The PSS [28] is a widely-used measure of stress perception that demonstrates good internal consistency (α = 0.68 to 0.78) and convergent validity including prediction of anxiety and anger (β = 0.54–0.68) [32]. Total PSS will be the variable analyzed from this measure. The CBI is a measure of burnout with separate personal, work, and client-related burnout subscales [29]. Psychometric properties include high internal consistency (Cronbach’s α = 0.85 to 0.87) and construct validity (negative correlations with measures of mental health and vitality; *r* = 0.39–0.75) [30]. Work- and client-related burnout will be the primary variables of focus. The CES-D is a common tool for assessing depressive symptoms^31^ which shows convergence validity with clinical diagnosis of depression at r = .45, internal consistency of α = 0.82, and test-retest reliability of r = 0.52 [33]. Total CES-D will be the primary analytic variable. The GAD-7 is a common tool for screening general anxiety symptoms^33^ which shows sensitivity and specificity for clinical diagnosis of anxiety above.80. Internal consistency is α = 0.92 and intraclass correlation is r = 0.83 [31]. Total GAD-7 will be the primary analytic variable. Data will be collected directly from participants via Qualtrics, with automatic calculation to reduce potential for error.

#### Statistical analysis, sample size, and power estimates.

Medium-to-large effect sizes [34] are expected based upon prior work using many of the same outcome assessments work [11,12]. The anticipated sample size of 200 students accounts for expected attrition of approximately 25%. To examine initial response to Unburdened –Student Edition, descriptive statistics will be utilized for Reaction variables, while other Learning, Behavior, and Results will be examined in condition over time using repeated measures analysis of variance. Extended follow-up through 12 months will be examined using latent growth curve models, an analytic technique within the structural equation modeling framework to examine change over time. Additionally, several potential predictors of outcomes will be explored via path analysis, including contribution of demographics (e.g., age, gender, etc.), program status (i.e., DVM versus RVT), and employment status/number of hours worked (e.g., part- versus full-time). Mode of analysis will be intent-to-treat (primary) and per protocol (secondary). We will use multiple imputation to address attrition in the intent-to-treat objective.

#### Data and safety monitoring plan.

A licensed Psychologist (PhD) who is knowledgeable about human subjects research and study methodology, and free from conflicts of interest has agreed to serve as an independent safety officer for this study. This individual has reviewed the study protocol and will monitor study enrollment, any adverse events, and IRB compliance. Reports outlining study progress with recruitment, retention, and adverse events will be submitted to the safety officer at 6-month intervals throughout the trial (or, in the case of a serious adverse event, as they occur).

#### Dissemination.

The plan for dissemination of this work considers sharing of study data, results, and materials. The trial is registered with Open Science Framework, where deidentified data and protocols will be uploaded following study completion. Results will be shared in peer-reviewed scientific journals and through team-led science blog sites. Finally, the intervention created for this work will be integrated into a single program that is accessible at the click of a link, and made available for use by DVM and RVT programs pending successful outcomes.

## Discussion

The significance of this study lies within the potential for Unburdened – Student Edition as a standardized way to help students preparing for the veterinary field learn tangible strategies for essential competencies such as reflection on personal actions (e.g., practicing self-reflection) and attention to personal well-being (e.g., recognizing sources of workplace stress and acting to remedy adverse situations). These skills will optimally translate to safeguard the emerging workforce in veterinary medicine against psychological distress related to a pervasive occupational hazard in the field of veterinary medicine (i.e., difficult client interactions). If successful, the program will be made available for DVM and RVT programs to embed into student curricula.
